# A population-based cohort to evaluate drug safety during pregnancy (PREGVAL): rationale, design, and baseline characteristics

**DOI:** 10.1007/s10654-025-01260-7

**Published:** 2025-06-23

**Authors:** Francisco Sánchez-Sáez, Gabriel Sanfélix-Gimeno, Isabel Hurtado, Aníbal García-Sempere, María Garcés-Sánchez, Fran Llopis-Cardona, Salvador Peiró, Clara L. Rodríguez-Bernal

**Affiliations:** 1 Health Services Research and Pharmacoepidemiology Unit, FISABIO, Valencia, Spain; 2 Spanish Network for Research on Chronicity, Primary Care, and Health Promotion (RICAPPS), Madrid, Spain; 3 Departament of Applied Statistics and Operational Research, and Quality, Universitat Politècnica de València, Valencia, Spain; 4 Dirección General de Salud Pública. Generalitat Valenciana [General Directorate of Public Health of the Valencian Community, Spain], Valencia, Spain

**Keywords:** Pregnancy, Drug safety, Perinatal pharmacoepidemiology, Outcomes, Real-world data, VID, Cohort profile

## Abstract

The main aim of the PREGVAL cohort is to evaluate and monitor the in-utero safety of medication administered to pregnant women, as usually this population is excluded from clinical trials assessing drugs. It is a population-based cohort, comprised by pregnant women and their offspring in the Valencia Region (Spain), between July 1st 2009 and December 31st 2021, which will be followed from 6 months pre-conception until death, loss of coverage or end of study, with over 520.000 pregnancies for the study period. The data source will be the Valencia Integrated Database (VID), which links records at the individual level using a unique personal identification code, with exhaustive data on comorbidities (diabetes, hypertension, etc.) and other clinical variables, sociodemographic variables (age, socio-economic status, risk of social exclusion) and other potential confounders (smoking, alcohol intake). It also provides accurate data on timing of exposure for most livebirths and all of the stillbirths occurred in the region, and partially for spontaneous abortions and elective terminations (due to the availability of gestational age by ultrasound or last menstrual period for these outcomes allowing the calculation of the date of conception) and allows the identification of medications prescription and dispensation data with a very high detail, avoiding the problem of potential exposure misclassification present in many of the existent registries/cohorts. Regarding outcomes, there is exhaustive and accurate data on pregnancy complications, spontaneous abortions, terminations (in this case, those occurred within the public health system), birth weight, preterm birth, congenital anomalies, and perinatal mortality; as well as the availability of post-natal long-term follow-up. These features make the PREGVAL study one of the largest and most exhaustive cohorts to assess real-world in-utero safety of medications to date.

**Supplementary Information:**

The online version contains supplementary material available at 10.1007/s10654-025-01260-7.

## Background and rationale

Pharmacotherapy use during the gestational period has rose progressively over the years [1, 2]. This is mainly attributed to the change in the demographics of pregnant women (there is a higher percentage of older pregnant women), the growth in the presence of pre-existing diseases and the increase in obstetric complications that require medications during pregnancy [1]. Despite this, pregnant women are frequently termed ‘therapeutic orphans’ as they are excluded from most randomized clinical trials of drug development and evaluation [3], largely due to ethical reasons (given the teratogenic potential of drugs). Given the relatively limited research and scientific information on drugs in pregnancy, clinicians have to treat this vulnerable population with insufficient data on efficacy and safety [4].

Important institutions such as the Center for Disease Control and Prevention (CDC) of the US, have acknowledged a clear lack of certainties regarding safety of drug use during pregnancy, and established the urgent need to address this information gaps [5].

Because of this, there is continuous interest in using electronic clinical databases that reflect routine clinical practice, and that allow assessing the safety and effectiveness of drugs in pregnancy [6]. In Europe, the usefulness of these databases in terms of drug use and safety was initially evaluated mainly in the context of EUROCAT (European network of population-based registries for the epidemiological surveillance of congenital anomalies) [7], [8]. Limitations of these databases have been acknowledged: (1) Regarding exposure: many do not have information on gestational age at delivery, which difficult the calculation of time of conception, and therefore timing of exposure might not be accurate. Some of them do not have precise data on medication exposure [7, 8]; (2) Regarding potential confounders: Few have data on smoking and alcohol consumption [7], which have long been established as important confounders when assessing any exposure and several pregnancy outcomes [9–13].

Birth registries from Nordic countries have been widely used to assess drug use or safety during pregnancy, being a reference for its overall quality and completeness [14]. These registries include data on all pregnancies resulting in the delivery of live born and stillborn infants from week 22 in Denmark, Finland, Iceland, and Sweden and week 12 in Norway [14, 15]. Information on abortions is not available in most of these registries [14]. The inability to identify early pregnancy loses and terminations incorporates selection bias into the results by excluding abortions/fetal loses that might be related to the exposure [6]. The linkage of birth registries with other registries made it possible to identify, through diagnostic codes, at least partially spontaneous abortions and terminations of pregnancy for fetal anomaly in the case of Denmark [16], and different types of early pregnancy losses in the case of Norway [17]. However, gestational age was not available for these outcomes and therefore, it had to be imputed in all of the cases [17].

Worldwide, studies on drug use or drug safety during pregnancy have been carried out in the last years, with an important production of evidence using US administrative databases based on claims of insured women. It has been recognized that misclassification and missing information are the main limitations of the US and other data sources [18–23]. Specifically, missing mother-infant linkages [21, 24–26], establishment of gestational age in an arbitrary manner [26–28] and lack of information on early losses [25, 29] and key confounders such as socioeconomic status [6, 30, 31] have been acknowledged. Furthermore, ascertainment of baseline comorbidities or outcomes is performed through diagnostic codes recorded in claims and use of a single diagnose is common but not appropriate as these codes might be used to justify procedures but not necessarily mean that the condition indicated by the code exist [6]. It is worth noting that important efforts have been made recently to overcome some of these limitations using administrative U.S. databases [19] and other datasources [32].

In other settings, there are examples such as a population-based cohort, assembled in China by using electronic records linkage to assess drug exposure during pregnancy. Its profile has been described very recently [33].

We have constructed a cohort (PREGVAL), by using data of the Valencian Health System Integrated Database - VID- (and their associated databases and registries) which is one of the most comprehensive and complete electronic routine clinical practice information systems in Spain, and probably in Europe [34], set-up in the context of a universal health coverage system in a population of around 5 million inhabitants [34]. The data covers the whole pathway of care (primary and specialist care, emergency room care and hospitalizations) and it is complemented with multiple exhaustive pregnancy-related registries (Metabolic Disease Registry -equivalent to a birth registry-, Congenital anomalies Registry, Perinatal mortality registry, etc.) [34].

All the information can be linked at the individual level through a single personal identification code, providing comprehensive individual-level data fed by all the databases from 2008 to date [34]. Also, mother to child linkage is provided by the Metabolic Diseases Registry, which allows the follow-up of the newborns into infancy and childhood. Furthermore, it is possible to identify not only live- and stillbirths, but also spontaneous abortions and partially, terminations of pregnancy (including those due to fetal anomalies). Many of these features are shared with the Nordic registries, including the size of the base population, which is similar to that of most Nordic countries [14]. The PREGVAL cohort has some distinctive features such as availability of both, prescribing and dispensing data from primary and specialist care with a very high detail, laboratory data from primary and specialist care, precise data on gestational age (calculated by ultrasound or last menstrual period -LMP) not only for live- and stillbirths but also for an important proportion of early pregnancy loses, making it possible to impute the gestational age for the remaining ones. Also, data on smoking and alcohol intake not only for live births and stillbirths but also for other pregnancy ends.

PREGVAL will provide valuable information on medication use and safety for pregnant women and their offspring, including the relationship with long-term outcomes such as child neurodevelopment.

The objective of the current paper is to present a profile of the participants of the PREGVAL cohort, including (i) a description of the methods used to assemble the cohort, (ii) characteristics of the population.

## Methods

### Design

Retrospective population-based cohort, comprised by pregnant women and their offspring, between July 1, 2009 and December 31, 2021 (the cohort is planned to be periodically updated), which will be followed from 6 months pre-conception until end of pregnancy (delivery, stillbirth, spontaneous abortion, elective termination), death, loss of coverage. This cohort does not include pregnancy planners per design. Elective terminations performed outside the public health system are not included either as this information is not available in VID. Exclusion criteria: non-residents in the Valencia region, due to limitations in the follow-up of the prescriptions and events foreseen in the study; women without pharmaceutical benefits covered by the Spanish NHS (civil servant service insurance mutualities) due to the limitations to identify their pharmaceutical consumption since it is not available in VID.

### Setting

The study was carried out in the Valencia region, Spain and, specifically, in the population covered by the Valencia Health System (VHS), the public health system that covers 97% of the population of the region, around 5 million inhabitants.

### Data sources

**Fig. 1:**
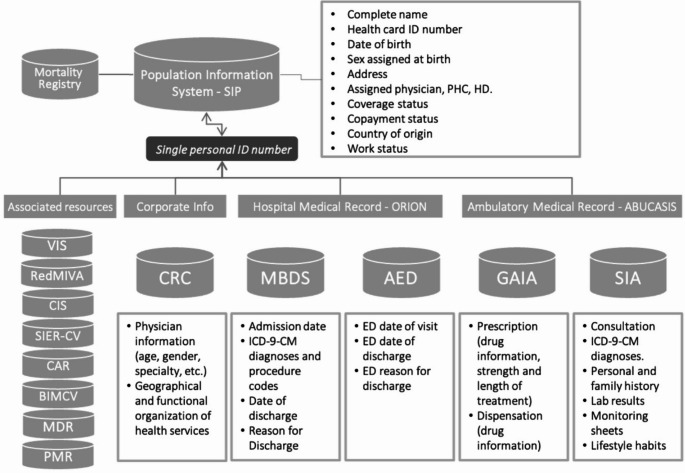
Valencia health system integrated database (VID) and their associated registries. Abbreviations: VIS, Vaccine Information System; RedMIVA, Microbiological Surveillance System; CIS, Cancer Information System; SIER-CV, Rare Diseases Information System; MDR, Metabolic Diseases Registry; PMR, Perinatal Mortality Registry; CAR, Congenital Anomalies Registry of the Valencia Region; BIMCV, Medical Image Bank; MBDS, Minimum Basic Data Set; AED, Accident & Emergency Department; CRC, Catalogue of Corporate Resources; GAIA, Pharmaceutical Module; SIA, Ambulatory Information System. Adapted from: Sanfélix-Gimeno, BMJ Open 2015; Garcia-Sempere, Int J Epidemiol, 2020. [Creative Commons Attribution Non Commercial (CC BY-NC 4.0) license].

The necessary information was obtained from VID for the time window January 1, 2009 to December 31, 2021. This way we obtained all the information (baseline-lookback period of 6 months, which allows to have also information of drug exposure available from preconception and throughout pregnancy) of all women included in the 2009–2021 cohort. The information was obtained specifically from: (1) Population Information System (SIP) that provides a unique patient identification number (included in all health information systems) and registers some sociodemographic characteristics (e.g., age, gender, country of origin, income, etc.), residence and coverage information (including the date of death); (2) Minimum Basic Data Set (MBDS) at hospital discharge that includes diagnoses and procedures under hospitalization coded by the International Classification of Diseases 9th revision Clinical Modification (ICD9CM) until 2015 and by the ICD10ES since 2016; in addition to key information for the present study such as gestational age and weight of newborns; (3) Ambulatory Information System (SIA), the electronic medical record available in all primary care centers and specialized outpatient care that includes information on patients regarding active diagnoses, personal and family medical history, laboratory results, lifestyle habits, results of diagnostic tests, etc., as well as health service utilization; and all the information on both prescriptions issued by primary care doctors and specialists and the prescriptions filled by the patients. (4) Electronic obstetric sheet (EOS). This is actually contained in SIA but we are briefly describing it separately giving its relevance in the pregnancy stage. EOS is mainly filled out by midwifes, containing information recorded at every prenatal visit from the first pregnancy-related appointment. It contains, among others, date of LMP and/or gestational age by ultrasound, maternal lifestyle factors during pregnancy, end of pregnancy and type of end, (e.g. spontaneous abortion, elective termination, etc.) with date of occurrence; (5) Accident & Emergency Department (AED) clinical record, which provides triage data, diagnoses, tests and procedures performed in public emergency rooms; (6) Corporate Resources Catalog (CRC), includes information on both, geographical and functional organization of the provision of care in the region (distribution of hospitals, primary care centers, etc.) and health care professionals (including age, gender and specialty); (7) Metabolic diseases registry (MDR), which is actually a birth registry, including all live births of the region, from both public and private hospitals; (8) Perinatal mortality registry (PMR) which registers all perinatal deaths of the region, from the public and private health systems; (9) Vaccine information system (VIS); and 9) Congenital anomalies registry of the Valencia Region (CAR). See Fig. 1 for a graphic description of the Valencia Integrated database and associated registries.

### Pregnancy cohort construction

#### Algorithm to identify pregnancies

The algorithm is developed using a hierarchical approach where records with available gestational age are prioritized, allowing an accurate assessment of the duration of pregnancy. If a pregnancy is identified in one step, the interval of that pregnancy (+ 40 days at the end) is blocked for subsequent steps. Therefore, only pregnancies not identified in a previous step are collected in each step.

**Fig. 2:**
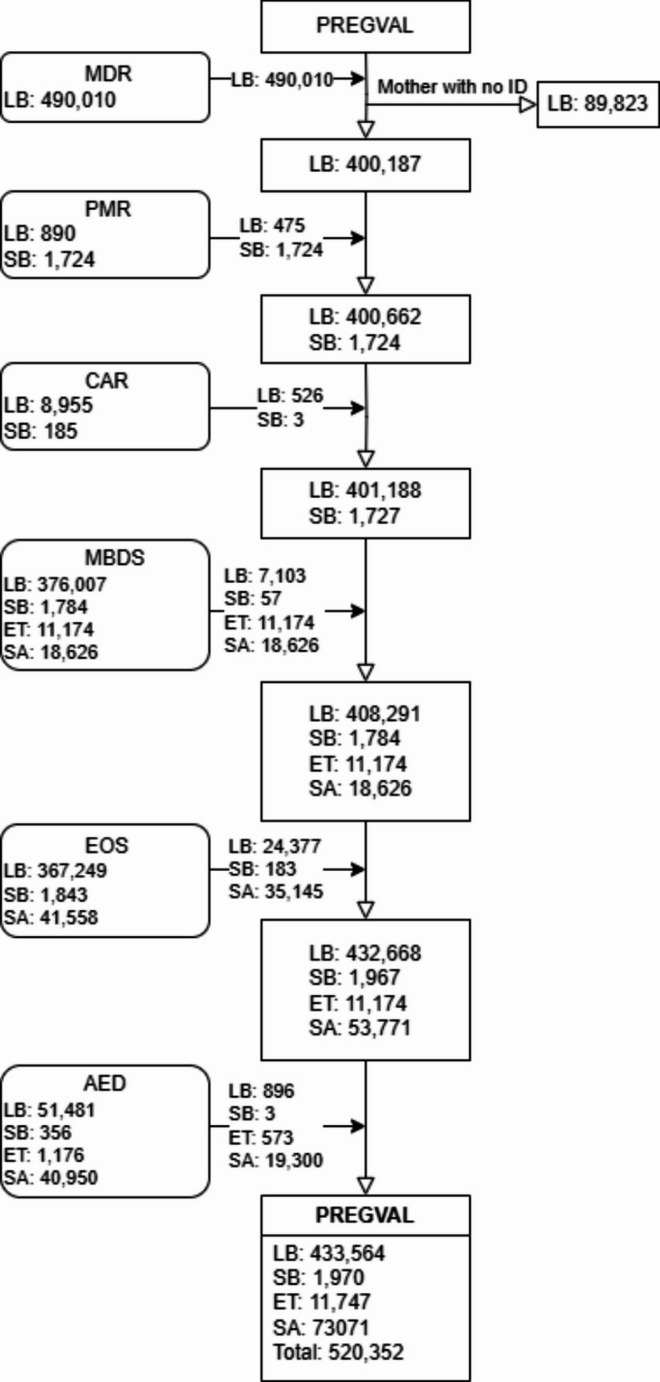
Cohort construction flowchart*. Abbreviations: MDR, Metabolic Diseases Registry; PMR, Perinatal Mortality Registry; CAR, Congenital Anomalies Registry of the Valencia Region; MBDS, Minimum Basic Data Set; EOS, Electronic Obstetric Sheet; AED, Accident & Emergency Department; LB, Live birth; SB, Stillbirth; ET, Elective termination; SA, Spontaneous Abortion. Only pregnancies not identified in a previous step are collected in each step. * Only pregnancies not identified in a previous step are collected in each step

The process starts with the collection of live births from the MDR, which records all live births with a high percentage of recorded gestational age (98%) and weight at birth (98%). However, there are errors or missing values for some of the mothers’ identifiers, so we were not able to retrieve all livebirths (linked to the mother) from this registry. Then some additional live births and most of the stillbirths are gathered from the PMR. Next, additional live births and stillbirths with birth defects are taken from the CAR. Then we use the MBDS to include elective, spontaneous and unknown abortions, as well as some live births and stillbirths that are missing from other registries. Then we collect additional data on live births, stillbirths and spontaneous abortions from the Electronic Obstetric Sheet (EOS), which is available in the SIA. Finally, for records from AED, pregnancies are retrieved using ICD-9 and ICD-10 codes. See Fig. 2. Given that pregnancies are identified sequentially, and pregnancy outcomes are determined based on the highest hierarchical source, following the order: MDR > PMR > CAR > MBDS > EOS > AED. In case of duplicates or discordant records in pregnancy outcomes, we applied a hierarchical reconciliation process. When conflicting outcomes were found across sources, the outcome recorded in the highest-ranked source (MDR > PMR > CAR, etc.) was prioritized.

#### Pregnancy period definition

In order to adequately estimate the pregnancy period (and therefore, the exposure period), an accurate definition of the conception date, as well as of the end of pregnancy date is of utmost importance. *Start of pregnancy* was defined as the conception date, *end of pregnancy* was defined as occurrence of spontaneous abortion, elective termination, stillbirth or live birth.

##### Date of conception

The following criteria was used to define conceptional date: (1) From gestational age (mainly calculated by ultrasound) available in the MDR, PMR, CAR, MBDS, EOS and AED. Currently this information is available in 97.5% of deliveries) (2) In the remaining deliveries, if weight is available it was estimated from the date of delivery (minus 40 weeks when the child’s weight is greater than 2.5 kg, and using a linear regression model in the case of lower weight children), this approach has been previously used by our group [35]; (3) In the case of stillbirths, gestational age as defined by ultrasound was available in the 97.4% of pregnancies. (4) In pregnancies that have ended in abortion, gestational age was used when available (50.4% of the cohort). Regarding elective terminations, gestational age is present in 9.6% of pregnancies. If information on gestational age was not retrieved, it was imputed using the median gestational age derived from the type of end of pregnancy.

##### End of pregnancy date

The date in which the pregnancy ended was defined as follows: (a) In the case of live birth: date of birth registered in MDR, PMR, CAR, MBDS, EOS or AED (b) In the case of stillbirth: the date in which the fetal death was registered (the date of occurrence of the death) in the PMR, CAR. MBDS, EOS or AED; (c) In the case of elective terminations: date in which the procedure was registered in the MBDS or AED; (d) In the case of spontaneous abortion: date in which the diagnostic code for spontaneous abortion was registered in the MBDS or AED, or that type of end was registered in EOS.

### Drug exposure information

Obtained from the pharmaceutical module (GAIA) of the ambulatory medical record. It contains all the information on both, prescriptions issued by primary care doctors or specialists and the prescriptions filled by the patients, including active ingredient, pharmaceutical presentation (dose, type of pharmaceutical forms, number of forms), dosage (prescribed dose and cadence), prescription and dispensing dates, etc. See supplementary Table 1 for a detailed description of prescription and dispensing data available.

### Outcomes

#### Pregnancy and neonatal outcomes

It is important to note that the PREGVAL cohort is not a birth cohort, but a pregnancy cohort (with its limitations, not including pregnancy planners), and thus, it retrieves data not only on livebirths but also on pregnancies which ended in stillbirth, spontaneous abortion or elective termination. Among live births, we considered the following outcomes: (a) Congenital anomalies: Retrieved from the congenital anomalies registry. All diagnoses have been validated, so there is a low likelihood of misclassification in information obtained from this registry. Information of birth defects diagnosis is collected from livebirths and stillbirths; (b) Low birth weight: Defined as birth weight under 2500 gr regardless of gestational age; and (c) preterm birth: Defined as birth before the 37th gestational week.

#### Long-term outcomes

Given that we are able to link the mother and offspring, follow-up from birth and along the lifecycle is feasible, to assess postnatal outcomes including (neuro) developmental disorders, among others.

### Covariates

A wide range of key factors that are related to relevant pregnancy outcomes and can confound the association with drug exposure are available, as follows: (1) *Concomitant medications during pregnancy*: Information is available for primary and specialist care, and it includes: active ingredient, pharmaceutical presentation (dose, type of pharmaceutical forms, number of forms), dosage (prescribed dose and cadence), prescription and dispensing dates, etc. (see supplementary Table 1); (2) *Sociodemographics*: maternal age, income level (copayment, which is based on personal income), risk of social exclusion as determined by a composite index; (3) *Chronic comorbidity* (See supplementary Table 2); (4) *Pregnancy-related diseases* (see supplementary Table 2); (5) Laboratory data, available for primary and specialist care. (6) *Lifestyle habits*: smoking, alcohol consumption, recreational drug abuse, sedentary behaviour (See supplementary Table 2). These variables were ascertained by diagnoses codes (which in our case are active diagnosis codes, that for chronic conditions, once they are activated remain activated); (7) *Previous medication use*: Availability of information as in the item “medications during pregnancy” (see supplementary Table 1). The time window can be extended backwards at least three months; (8) *Use of health services previous to conception date or during pregnancy*: visits to the emergency room, primary care, specialized care-identifying the specialty, hospitalizations); (9) *Healthcare system*: Basic Health Zone, Health Department.

## Key findings

### Cohort size

PREGVAL is a population-based cohort of over 520,000 pregnancies (Fig. 2) which ended in live births, stillbirths, spontaneous abortions and terminations of pregnancy (in this case, only those performed within the public health system). Its dynamic nature allows new pregnancies/women entering the cohort. It is foreseen that every two years, starting in 2025, the cohort incorporates new pregnancies.

### Main information available

The main information available regarding pregnancy-related variables, pregnancy, newborn, infant and child outcomes, drug exposure, sociodemographics, clinical and other variables in the PREGVAL cohort are presented in Table 1.

**Table 1:** Main information available in the PREGVAL cohort, 2009–2021

Category	Examples of variables
Pregnancy-related variables/data	-LMP -Gestational age by ultrasound -Pregnancy-related diseases (diagnosis of gestational hypertension, gestational diabetes, preeclampsia, etc.) -Reproductive history (parity, previous pregnancy losses, etc.)* -Antenatal care visits -Type of delivery (vaginal, C-section)
Pregnancy and neonatal outcomes	-Spontaneous abortion -Termination of pregnancy (including due to birth defects)** -Stillbirth -Livebirth -Perinatal death -Major congenital anomalies such as heart defects and orofacial clefts -Minor congenital anomalies -Preterm birth -Low birth weight -Birth weight
Infant, child and long-term outcomes	As we are able to link mother and offspring data, and offspring will also belong to the public health system, follow-up after birth is possible. Data such as neurological development, growth, and incident illness based on diagnostic codes will be available for infants and children. Overall, follow-up from birth and up to adolescence is feasible in this cohort.
Drug exposure during pregnancy	-All prescriptions issued by physicians and exact dates -Dispensation of medicines and exact dates (can be linked to prescriptions) -Detailed data such as active ingredient, pharmaceutical presentation (dose, type of pharmaceutical forms, number of forms), dosage (prescribed dose and cadence)
Previous drug exposure	-All prescriptions issued within the previous year -All dispensations within the previous year -Detailed data as above
Socio-economic status and demographic variables*	- Age -Country of origin -Income -Risk of social exclusion
Clinical variables	-All active diagnosis registered by physicians (ICD9CM, ICD10CM) such as diabetes, hypertension, depression, anxiety, obesity, asthma, etc.* - Family medical history -Lab results and other measurements for primary and specialist care such as blood pressure measurements, glycemic control, etc. -Health services utilization -Emergency visits and diagnoses
Other maternal variables registered by the physician*	-smoking -alcohol intake **-**drug abuse

*Data obtained at baseline (6 months lookback period)

** Only those performed within the public health system

### Baseline characteristics

Table 2 shows the baseline characteristics of the PREGVAL cohort. We identified 520,352 pregnancies during the study period (See supplementary Table 3 for number of pregnancies identified per year). Mean age of the cohort was 31.9 year, 27.6% were foreigners and 21.2% were at risk of social exclusion. Regarding clinical variables, 2.0% had chronic hypertension, 1.4% diabetes, 6.5% depression and 5.5% obesity when entering pregnancy.

**Table 2:** Baseline characteristics of the PREGVAL cohort, 2009–2021

Characteristic	*N* = 520,352	%
***Maternal age, years***		
Mean age (SD)	31.89	5.81
***Age (categories)***		
≤ 25	81,088	15.58
26–35	313,188	60.19
> 35	126,076	24.23
***Country of origin***		
Spain	376,616	72.38
Other European	36,436	7.00
Others	99,714	19.16
Unknown	7,586	1.46
***Income***		
< 18.000 euros	378,539	72.75
18.000-100.000 euros	87,347	16.79
> 100.000	565	0.11
No income	25,463	4.89
Unknown	28,438	5.47
***Risk of social exclusion***		
*Yes*	110,237	21.19
***Lifestyle habits***		
Smoking	38,420	7.38
Alcohol use	3,032	0.58
Sedentary behaviour	97	0.02
Drug abuse	4,624	0.89
***Selected Chronic diseases***		
Hypertension	10,127	1.95
Congestive heart failure	118	0.02
Lipid disorders	39,720	7.63
Diabetes	7,312	1.41
Depression	33,903	6.52
Anxiety	130,266	25.03
Psychotic disorder	954	0.18
Bipolar disorder	838	0.16
Obesity	28,380	5.45
Asthma	27,850	5.35
Epilepsy	3,204	0.62
**Drug use previous to pregnancy**		
Pregnancies with at least one prescription dispensed in the three-month period before conception	216,043	41.52

### Pregnancy and birth characteristics

Table 3 shows the characteristics during pregnancy of the PREGVAL cohort.

**Table 3:** Pregnancy and birth characteristics, PREGVAL cohort, 2009–2021

Characteristic	*N*	%
***Pregnancy-related diseases***		
Gestational hypertension	5,150	0.99
Preeclampsia	7,299	1.40
Gestational Diabetes	35,878	6.89
**Pregnancy and neonatal outcomes**		
Spontaneous abortion	73,071	14.04
Elective termination	11,747	2.26
Stillbirth	1,970	0.38
Live birth	433,564	83.32
Perinatal death	2,199	0.42
Major congenital anomalies	8,945	1.72
Birth weight (mean, SD)	3,180.49	541.96
LBW	31,855	8.10
Preterm birth	30,707	7.08
Type of delivery*		
C-section	101,383	23.38
**Drug use during pregnancy**		
At least one drug prescribed/one prescription during pregnancy	432,468	83.11
At least one dispensed drug during pregnancy	413,238	79.42
Number of different drugs used (median, P25; P75)	2.00	1; 4

*For live births

Of the whole cohort, 6.9% had diagnosis of gestational diabetes, 1.0% gestational hypertension and 1.4% preeclampsia. Prevalences of pregnancy outcomes were: 1.7% congenital anomalies; 14.0% spontaneous abortion; 2.3% elective termination; 0.4% stillbirth; 83.3% live birth, and from them, 7.1% were born preterm and 8.1% had low birth weight. Regarding drug use, we found that 79.4% of the cohort had at least 1 medicine dispensed (84.9% for Live Births). Median number of active ingredients dispensed was 2.0 (P25:1, P75:4). The most used ATC’s were: B03 antianemics 28%, G03 sex hormones and modulators of the genital system 9.6%, and J01 antibacterials 9.5%.

### Strengths and weaknesses

(1) The large size of the cohort, which allows to study more accurately infrequent outcomes. However, it could be not large enough to study very rare ones. (2) availability of data on medication use prescribed in primary as well as specialist care during pregnancy (a group of patients that is not normally included in clinical trials) in a population of approximately 5 million inhabitants and over a long period of time (3) the high quality, exhaustiveness and completeness of the prescription-dispensing databases (4) The availability of uncommon data in studies of in-utero safety, useful for specific sub-studies within our cohort. i.e.: blood pressure or glycated hemoglobin measurements, which will allow the identification of controlled vs. uncontrolled women regarding diabetes or hypertension, among others. In the specific case of lab results, information is available for all patients at the outpatient setting (that is, primary and specialist care) for whom laboratory tests have been performed. (5) the possibility, thanks to the combination of different VID databases, of extensively characterizing the pregnant women participating in the study, and therefore also accounting for several important potential confounders, including smoking and alcohol intake which are available not only for live- and stillborn infants but also for abortions and terminations retrieved. It is important to note that this information comes currently from diagnostic codes, underestimating the true prevalence of smoking, we will work on adding in the near future another source of information to improve our estimation. Other characteristics such as age and country of origin coincide with official statistics of the region (6) The possibility to capture pregnancies that end not only in live and stillbirth but in spontaneous abortion and partially, in voluntary interruption including those due to fetal anomalies (we retrieve voluntary interruptions performed in the public health system only) (7) The availability of accurate gestational age (as measured by ultrasound or date of last menstrual period) not only for live births and stillbirths, but also in more than half of spontaneous abortions, given that this information, specially the latter is extremely rare in other databases at the global level, including those whose quality and completeness of data have been widely acknowledged. This feature allowed us to impute gestational age of the remaining ones using methods previously validated (8) The dynamic nature of the cohort, with updates foreseen every two years, which will permit that the follow-up time can actually be potentially along the lifecycle if needed, as long as the participants do not leave the region, also the data recency, as when a data extraction occurs, it contains data of up to the week before the date of extraction. (9) the extensive experience of our group in the management of large cohorts using real world data (RWD) for pharmacoepidemiological studies of safety and effectiveness, as well as the specific experience of some of its members, who have worked with cohort studies of pregnant women. (10) the possibility of ongoing monitoring on the intrauterine safety and effectiveness of medications administered to pregnant women.

Among the limitations, the following should be noted: (1) There is no prescription for drugs administered outside the VHS. This, although marginal for most prescription drugs, will tend to underestimate drug use, and it is difficult to quantify its impact; (2) In VID, data quality and completeness is, overall, very high, although some specific items may be of lower quality and variable between centers and over time. This problem is always present in RWD studies, and in our setting it is expected to have low impact, given the exhaustiveness of the data sources and the overall low percentage of missing values, although a certain level of misclassification or selection bias may potentially occur; (3) a marginal proportion of prescriptions in the VHS (less than 1%) do not use electronic prescription systems but manual prescriptions, meaning this information is not captured; (4) the coding of diagnoses in the MBDS changed–as of January 1, 2016 – to ICD-10-CM. Beyond the coding problems that may have occurred during the adaptation period, the ICD-9-CM and ICD-10-CM codes do not have a direct and unequivocal relationship and it is possible that the time series may be altered by changes in the coding. (5) The limited availability of gestational age in elective terminations retrieved: we were unable to retrieve gestational age data for most ET, therefore we have imputed it by using a previously validated method. Availability of gestational age for elective terminations is extremely rare in already existing databases or cohorts assessing drug safety in pregnancy. (6) Our cohort does not include pregnancy planners given the difficulty to retrieve this data from electronic databases, especially when pre-conceptional visits utilization is very low in the population. However, it includes all conceptuses that end in stillbirth and most conceptuses that end in livebirth in the whole Valencia Region, plus all abortions and terminations within the public health system. It is important to note that in our setting, most terminations (90%) are performed in the private health system, and therefore the prevalence shown for this specific outcome might be lower than that of the population.

## Concluding remarks

The PREGVAL cohort will provide valuable information on medication use as well as medication safety for pregnant women and their offspring, including the relationship with long-term outcomes such as child neurodevelopment, among others. Its distinctive features such as the comprehensive and detailed information on drug prescribing and dispensing, availability of laboratory data from primary and specialist care as well as data on potential confounders (socio-economic status, lifestyle habits, etc.) for all types of pregnancy end (and not only live- or stillbirths) identified, makes it a useful resource, adding to the already existing data sources, to generate evidence regarding drug exposure for this vulnerable dyad.

We foresee to use the PREGVAL cohort for the following purposes overall: (1) Monitoring real-world pharmacologic management of pregnant women (we will assess: drug prescription rates, by therapeutic group and trimester of pregnancy; time of medication exposure (by therapeutic groups) and for each trimester of pregnancy; prevalence of pregnancy outcomes such as: low birth weight, preterm delivery, spontaneous abortion, congenital anomalies (major and minor), and fetal and neonatal death; temporal trends of medication exposure during pregnancy; temporal trends of pregnancy outcomes rates). (2) Assessment of the safety of relevant medications with inconclusive or incomplete (present only for certain outcomes) evidence such as antihypertensives, new oral antidiabetics, medications to treat mental health conditions, among others.

## Electronic supplementary material

Below is the link to the electronic supplementary material.

Supplementary Material 1
