# Supplementary material for: A population-based cohort to evaluate drug safety during pregnancy (PREGVAL): rationale, design, and baseline characteristics
Source: Eur J Epidemiol. 2025 Jun 23;40(7):859–69. doi: 10.1007/s10654-025-01260-7 (PMC12304015; doi:10.1007/s10654-025-01260-7)
Supplement: Supplementary file 1 — Supplementary Material 1 [file 10654_2025_1260_MOESM1_ESM.docx]

**Supplementary material.**

European Journal of Epidemiology.

**A population-based cohort to evaluate drug safety during pregnancy (PREGVAL): rationale, design, and baseline characteristics.**

Francisco Sánchez-Sáez^1,2,3^, Gabriel Sanfélix-Gimeno^1,2^, Isabel Hurtado^1,2^, Aníbal García-Sempere^1,2^, María Garcés-Sánchez^4^, Fran Llopis-Cardona^1^, Salvador Peiró^1,2^, Clara Rodríguez-Bernal^1,2^*.

1. Health Services Research and Pharmacoepidemiology Unit, FISABIO, Spain.
2. Spanish Network for Research on Chronicity, Primary Care, and Health Promotion (RICAPPS), Spain.
3. Departament of Applied Statistics and Operational Research, and Quality, Universitat Politècnica de València, Valencia, Spain
4. Dirección General de Salud Pública. Generalitat Valenciana [General Directorate of Public Health of the Valencian Community, Spain], Spain.

***corresponding author:** Clara Rodríguez-Bernal. E-mail: [clara.rodriguez@fisabio.es](mailto:clara.rodriguez@fisabio.es)

Supplementary table 1. Availability of information regarding medication in the PREGVAL cohort.

| **Information regarding medication*** | | | |
| --- | --- | --- | --- |
|  | **Prescribing data** | **Dispensing data** | **Comments** |
| **Setting** | Primary and specialist care | Primary and specialist care |  |
| **Grannularity** | Active ingredient, pharmaceutical presentation and product | Active ingredient, pharmaceutical presentation and product |  |
| **Drug classification** | ATC and national code | ATC and national code |  |
| **Pharmaceutical presentation** | Dose, type of pharmaceutical forms, number of forms, administration route | Dose, type of pharmaceutical forms, number of forms, administration route | e.g., ENALAPRIL 5 MG / 60 TABLETS, ORAL |
| **Date of treatment initiation** | Yes | No | In the prescribing module, date of initiation and end of treatment is registered by the physician, allowing for the estimation of the duration of each treatment episode. |
| **Date of treatment**  **finalization** | Yes | No |  |
| **Dosing schedule** | Prescribed dose and cadence | No | e.g., ENALAPRIL 5 MG / 60 TABLETS, ORAL, with a dosing schedule of one tablet every 12 hours as defined in the prescription, for 30 days covered with medication) |
| **Date prescription/ dispensation** | Yes | Yes | Format: YYYY-MM-DD |
| **Indication** | Yes | No | ICD-9-CM/ICD-10-CM |
| **Linkage** | Yes | Yes | Ability to link each particular prescription with its dispensation with a unique identifier at the individual patient level. |
| **Completeness** | 100% | 100% | Errors or missing values are possible for Dosing schedule and Indication (2.9% and 3.2%, respectively). |

*Information retrieved from the Valencia Integrated Database (VID).

Supplementary table 2. Diagnostic codes used to identify lifestyle characteristics, selected chronic diseases and pregnancy-related diseases in the PREGVAL cohort.

| ***Characteristics*** | **ICD9CM** | | | **ICD10ES** |
| --- | --- | --- | --- | --- |
| ***Lifestyle habits*** | | | | |
| Smoking | | 305.1, 649.0*, 989.84, V15.82 | F17.*, T65.2*, Z72.0, Z87.891, O99.33* | |
| Alcohol use | | 291.*, 303.*, 305.0*, 357.5, 425.5, 571.0, 571.1, 571.2, 571.3, 980.0, E860.0 | E52.*, F10.*, G31.2, G62.1, G72.1, I42.6, K29.2, K70.*, K85.2*, K86.0, O35.4*, O99.31*, T51.*, Z71.41 | |
| Sedentary behaviour | | V69.0 | Z72.3 | |
| Drug abuse | | 292.*, 304.*, 305.2*, 305.3*, 305.4*, 305.5*, 305.6*, 305.7*, 305.8*, 305.9*, 648.30*, 655.5*, | F11.*, F12.*, F13.*, F14.*, F15.*, F16.*, F18.*, F19.*, O35.5*, O99.32* | |
| ***Selected Chronic diseases*** | | | | |
| Hypertension | 401.*, 402.*, 403.*, 404.*, 405.*, 437.2, 642.0*, 642.1*, 642.2* | | | I10, I11.*, I12.*, I13.*, I15.*, I16.*, I67.4, O10.* |
| Congestive Heart Failure | 398.91, 402.01, 402.11,  402.91, 404.01, 404.03,  404.11, 404.13, 404.91,  404.93, 425.4, 428.* | | | I09.81, I11.0, I13.0, I13.2, I42.0, I50.* |
| Lipid Disorder | 272.* | | | E78.* |
| Diabetes | 249.*, 250.*, 648.01, 648.03 | | | E08.*, E09.*, E10.*, E11.*, E13.*, O24.0*, O24.1*, O24.3* |
| Depression | 296.2*, 296.3*, 296.82, 298.0, 300.4, 301.12, 311 | | | F32.*, F33.*, F34.1, F41.8, F53.0, O90.6 |
| Anxiety | 293.84, 300.0*, 300.2* | | | F06.4, F40.*, F41.* |
| Psychotic disorder | 295.*, 297.*, 298.*, 299.* | | | F20.*, F21, F22, F23, F24, F25.*, F28, F29, F53.1, F84.* |
| Bipolar disorder | 296.0*, 296.1*, 296.4*, 296.5*, 296.6*, 296.7*, 296.80, 296.89, 296.9* | | | F30, F31* |
| Obesity | 278.00, 278.01, 278.03, 649.1*, 649.2*, V45.86, V85.3*, V85.4* | | | E66.0*, E66.1, E66.2, E66.8, E66.9, O99.21*, O99.84*, Z68.3*, Z68.4*, Z98.84 |
| Asthma | 493.* | | | J45.* |
| Epilepsy | 345.* | | | G40.* |
| ***Pregnancy-related diseases*** | | | | |
| Gestational hypertension | 642.3* | | | O13.* |
| Preeclampsia | 642.4*, 642.5*, 642.6*, 642.7* | | | O11.*, O14.*, O15.* |
| Gestational Diabetes | 648.8* | | | O24.4*, O99.81* |

ICD-9-CM: International Classification of Diseases, 9th Revision, Clinical Modification; ICD-10-CM: International Classification of Diseases, 10th Revision, Clinical Modification; include all category numbers after the “*”.

Supplementary table 3. Pregnancies identified in the PREGVAL cohort, by year*.

| **Pregnancies identified by year** | | | | | | | | | | | | | |
| --- | --- | --- | --- | --- | --- | --- | --- | --- | --- | --- | --- | --- | --- |
| **2009**** | **2010** | **2011** | **2012** | **2013** | **2014** | **2015** | **2016** | **2017** | **2018** | **2019** | **2020** | **2021***** | **2009-2021** |
| 25,364 | 51,693 | 49,344 | 47,287 | 46,213 | 45,688 | 45,220 | 43,430 | 42,026 | 39,459 | 38,216 | 34,732 | 11,680 | 520,352 |
| *Note that PREGVAL is a dynamic cohort and more pregnancies are forseen to be added every 2 years **Only pregnancy initiations from July 1^st^ were accounted for; ***Only pregnancies with conception and end date within 2021 were included. | | | | | | | | | | | | | |
